# Automated computation and analysis of accuracy metrics in stereoencephalography

**DOI:** 10.1016/j.jneumeth.2020.108710

**Published:** 2020-07-01

**Authors:** Alejandro Granados, Roman Rodionov, Vejay Vakharia, Andrew W. McEvoy, Anna Miserocchi, Aidan G. O'Keeffe, John S. Duncan, Rachel Sparks, Sébastien Ourselin

**Affiliations:** a School of Biomedical Engineering and Imaging Sciences, King's College London, UK; b National Hospital of Neurology and Neurosurgery, London, UK; c Dept of Clin and Experim Epilepsy, UCL Queen Square, Inst of Neurol, UK; d Dept of Statistical Science, University College London, UK

**Keywords:** Epilepsy, SEEG, Accuracy metrics

## Abstract

**Background:**

Implantation accuracy of electrodes during neurosurgical interventions is necessary to ensure safety and efficacy. Typically, metrics are computed by visual inspection which is tedious, prone to inter-/intra-observer variation, and difficult to replicate across sites.

**New Method:**

We propose an automated approach for computing implantation metrics and investigate potential sources of error. We focus on accuracy metrics commonly reported in the literature to validate our approach against metrics computed manually including entry point (EP) and target point (TP) localisation errors and angle differences between planned and implanted trajectories in 15 patients with a total of 158 stereoelectroencephalography (SEEG) electrodes. We evaluate the effect of line-of-best-fit approaches, EP definition and lateral versus Euclidean distance on metrics to provide recommendations for reporting implantation accuracy metrics.

**Results:**

We found no bias between manual and automated approaches for calculating accuracy metrics with limits of agreement of ±1 mm and ±1°. Automated metrics are robust to sources of errors including registration and electrode bending. We observe the highest error in EP deviations of μ = 0.25 mm when the post-implantation CT is used to define the point of entry.

**Comparison with Existing Method(s):**

We found no reports of automated approaches for quality assessment of SEEG electrode implantation. Neither the choice of metrics nor the possible errors that could occur have been investigated previously.

**Conclusions:**

Our automated approach is useful to avoid human errors, unintentional bias and variation that may be introduced when manually computing metrics. Our work is relevant and timely to facilitate comparisons of studies reporting implantation accuracy.

## Highlights

• Automatic computation of SEEG accuracy metrics agree with those done manually.
• The choice of image to generate a scalp model has an effect on entry point metrics.
• Metrics have the lowest mean and variability when using an electrode bolt axis.
• Lateral shift deviation should include a measure of insertion depth error.

## 1 Introduction

Stereoelectroencephalography (SEEG) is a stereotactic neurosurgical procedure for estimating the Epileptogenic Zone (EZ) in patients with focal refractory epilepsy (Mullin et al., 2016). It consists of stereotactically implanting multiple electrodes, typically between 5 and 18 (Jayakanar et al., 2016), into brain areas that are selected by a multidisciplinary clinical team based on multi-modal investigations. SEEG allows a three-dimensional definition of the EZ and is appropriate for exploration of sulcal and deep cortical areas (Jayakanar et al., 2016; Minotti et al., 2018). Accuracy of SEEG electrode implantation is crucial (a) to ensure safety by avoiding damage of intracranial blood vessels, and (b) to guarantee sampling of the suspected EZ (especially near small structures, e.g. posterior hippocampus).

SEEG implantation accuracy is affected by several physical and physiological processes that can result in entry point (EP), target point (TP), and angle displacements, between planned and implanted trajectories. When investigating implantation error, it is important to distinguish between physical implantation errors and errors in our ability to measure differences. Among those factors which cause implantation errors include: 1) misregistration between planning and navigation scans, 2) registration accuracy of neuronavigation system (ideally below 0.5 mm), 3) positioning of drill guide arm (either robotic or manual), 4) motion of scalp relative to skull (which could cause misalignment of entry arm), 5) drilling errors (instability, slipping), 6) electrode deviations affected by surgical technique (use of stylet or not), structural and biomechanical properties of soft tissue (heterogeneity, angle when crossing tissue interfaces), mechanical properties of electrodes, and 7) post-implantation physiological response (Cerebrospinal fluid (CSF) leak, tissue swelling). Our ability to measure implantation errors can be affected by: 1) registration inaccuracies between post-operative and navigation scans, 2) bolt movement (potentially related to skull thickness) after surgery, and 3) inaccuracies of SEEG electrode segmentation (particularly using automated approaches) of the contacts and bolt. Some implantation errors are controlled by surgical implantation technique (registration, neuronavigation system, positioning device, drill) whereas others are present regardless of technique (CSF loss, tissue response to electrode).

Previous studies have described SEEG implantation accuracy qualitatively and quantitatively, mainly when comparing 1) positioning technique (robotic system versus mechanical arm), 2) stereotactic frames versus frameless image-guided systems, and 3) customised patient-specific fixtures (Vakharia et al., 2017). Qualitatively, neurophysiologists use post-implantation CT images co-registered to MRI to examine the position of electrodes relative to the cortical and subcortical structures and decide which anatomical brain structures are part of the seizure onset zone (Nowell et al., 2014). Accuracy of SEEG electrode implantation is typically reported quantitatively as distance metrics, as either Euclidean or lateral shift (see Table. 1). Most studies only report TP localisation errors and of those studies reporting EP localisation errors, the position of entry points and the estimation of implanted trajectories are often not clearly defined. For instance, Cardinale et al. (2013) use the “major axis of implanted electrode” not explaining how it was estimated. Due to heterogeneity of the metrics used, comparison across studies becomes limited (Vakharia et al., 2017). Moreover, the interval before a post-implantation CT scan is taken after surgery is rarely reported, despite evidence that CSF loss can influence bending of electrodes (Lalys et al., 2014). Lastly, previous studies report metrics computed using measurements mostly acquired by visual inspection, resulting in a process that is tedious and prone to inaccuracies and bias.

**Table 1:** Accuracy metrics of SEEG electrode implantation reported in the literature.

Study	Distance Metric	Metrics	Where is EP defined?	Where is TP defined?	Implanted Trajectory	Post-op Image (time)
Balanescu et al. (2014)	Lateral Euclidean	EP TP Depth	Anchor bolt	most distal contact	N/A	CT (not stated)
Cardinale et al. (2013)	Euclidean	EP TP	Interior skull surface	most distal contact	LBF of electrode	Oarm (end of surgery)
Davies et al. (1999)	Euclidean	TP	N/A	contact position closer to hippocampus	N/A	MR (not stated)
Dorfer et al. (2017)	Euclidean	EP TP	Skull level	most distal contact	N/A	CT (not stated)
(Gonzlez-Martnez et al. (2016)	Euclidean	EP TP	Cortical surface	most distal contact	N/A	CT (not stated)
Hou et al. (2014)	Euclidean	TP	N/A	most distal contact	N/A	CT (end of surgery)
Mascott (2006)	Euclidean	TP	N/A	most distal contact	N/A	MR (within 24 h after surgery)
Mehta et al. (2005)	Euclidean	TP	N/A	centre of hypointense signal	N/A	MR (not stated)
Munyon et al. (2013)	Euclidean	TP	N/A	centroid	N/A	CT (not stated)
Nowell et al. (2020)	Lateral	TP Depth	N/A	most distal contact	Bolt axis	CT (within 4 h after surgery)
Rodionov et al. (2019)	Lateral	EP TP Angle Depth	Scalp level	most distal contact	Bolt axis	CT (within 4 h after surgery)
Roessler et al. (2016)	Euclidean	TP	Skull level	most distal contact	N/A	iMR (N/A)
Verburg et al. (2016)	Euclidean	Depth	N/A	last hyperdense signal	N/A	CT (not stated)

## 2 Contributions of this paper

Our main motivation is to automatically compute electrode implantation accuracy relative to planned trajectories within a platform that supports all stages of electrode implantation, from planning to post-surgical assessment. We focus on accuracy metrics commonly reported in the literature to validate our automated approach against metrics computed manually. We evaluate the effect of line-of-best-fit approaches, EP definition and lateral shift versus Euclidean distance on metrics to provide recommendations for reporting implantation accuracy metrics. We validate our approach in 15 patients implanted with a total of 158 SEEG electrodes Ad-Tech Med Instr Corp, USA).

## 3 Methods

### 3.1 SEEG electrode implantation assessment workflow

**Fig. 1:**
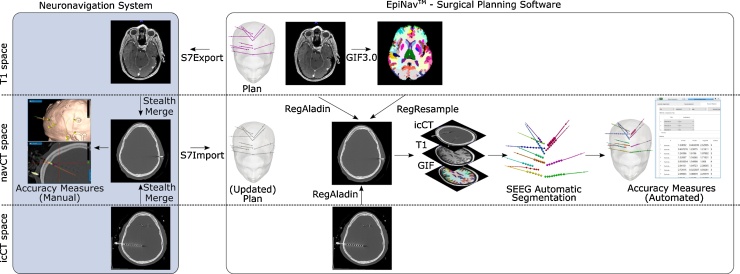
SEEG Electrode Implantation Assessment Workflow. T1-weighted MRI (T1), navigation CT (navCT) and post-implantation CT (icCT) images are acquired before, during and after surgery, respectively. An SEEG electrode implantation plan is created in EpiNav™ and exported to the neuronavigation system, where it may be updated by the operating neurosurgeon just before surgery. In the standard clinical workflow, accuracy measures are computed manually on the neuronavigation software in the navCT space. The surgical planning software EpiNav™ is used to compute accuracy measures automatically, where co-registration of the T1 and icCT to the navCT is performed (NiftyReg (Modat et al., 2014)) before automatically segmenting SEEG electrodes (Granados et al., 2018).

Implantation planning is performed using a T1-weighted MRI (T1) with gadolinium enhancement as previously described (Rodionov et al., 2019). Planned trajectories are exported to a Medtronic, Inc. S7 StealthStation™, the navigation system used in our study (Fig. 1 left). On the day of surgery, bone fiducials are placed into the skull of the patient and a navigation CT (navCT) image is acquired. The T1 is aligned (co-registered) to the navCT using StealthMerge™. The operating neurosurgeon inspects the planned trajectories in the navCT space, and if necessary, makes adjustments. The electrodes are implanted as specified by the plan using a frameless system, a precision aiming device (Medtronic) (Nowell et al., 2014). In this study, Ad-Tech electrodes were used for implantation. Within four hours post surgery, a CT (icCT) image is acquired to assess whether the SEEG implantation resulted in any complications (Nowell et al., 2014).

To calculate electrode implantation accuracy planned trajectories are imported from the navigation system into EpiNav™ whereas implanted trajectories are automatically segmented following (Granados et al., 2018) from the T1 and icCT images rigidly co-registered to the navCT using NiftyReg (Modat et al., 2014) (Fig. 1 right). A parcellation of the brain is computed from the T1 using GIF 3.0 (Cardoso et al., 2015) and re-sampled to the navCT space. The resulting electrode segmentation was inspected and adjusted (as necessary) to ensure accurate contact positions.

### 3.2 Metric calculation

**Fig. 2:**
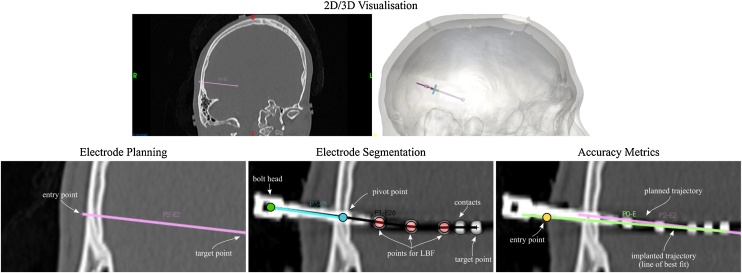
Estimation of SEEG electrode implantation accuracy measures. *Top:* 2D and 3D visualisation of electrode planning showing one electrode. *Bottom:* Close-up view of electrode planning on pre-operative CT image (*left*), segmentation on post-operative CT image (*centre*) and trajectories used for accuracy metrics (*right*). Automated segmentation identifies the position of the bolt head (green circle), pivot point (blue circle) and the position of contacts (black circles). A line of best fit (LBF) is computed for a number of contacts (red circles) and used to compute the position of the implanted entry point (yellow circle). EP and TP localisation errors and angle difference between implanted (green line) and planned (pink line) trajectories are computed. Manual computation of metrics is detailed in Sec. 3.4, whereas our proposed automated approach is detailed in Sec 3.3 as well as Appendix A and B.

We compute accuracy metrics (EP error, TP error, and angle difference) to measure how well an implanted trajectory, aligns with a planned trajectory (Fig. 2) (see Appendix A). Entry and target points are defined at the scalp level and at the position of most distal contact, respectively, similar to (Rodionov et al., 2019). Lateral shift is defined as the shortest distance between a point and a line, whereas Euclidean distance is defined as the shortest distance between two points, both metrics defined in 3D space (see Eq. A.1).

Two approaches to characterise the trajectory of an implanted SEEG electrode are computed: bolt axis and line of best fit. We define a line of best fit considering the first contact fully outside the implanted bolt through to all contacts within 20 mm distance of the first contact (see Appendix B).

### 3.3 Automated measurements

Bolt head, pivot point, and electrode contacts are computed via automated electrode segmentation (Granados et al., 2018). Automated metric computation is implemented in C++ using MITK (http://mitk.org), and the Eigen library (http://eigen.tuxfamily.org) with a graphics user interface in Qt. To execute our proposed software, a non-technical user loads the implanted electrode trajectories which were automatically identified (Granados et al., 2018), planned electrode trajectories, as well as a 3D mesh of the scalp. The automated computation of implantation accuracy metrics takes only few seconds to execute.

#### 3.3.1 Implanted entry point estimation

A surface mesh of the scalp is generated from the navCT image. The 3D mesh of the scalp and the implanted trajectory are used to define the implanted entry point (see yellow circle in Fig. 2).

#### 3.3.2 Implanted target point estimation

The position of the most distal contact is estimated after thresholding of the icCT image in navCT space (see pink circle in Fig. 2) followed by computing the centroid (Granados et al., 2018). Bolt axis and line-of-best-fit implanted trajectories are computed following Appendix B}. Given a list of planned and implanted trajectories, we automatically pair electrodes based on two criteria: 1) the closest distance between entry points, followed by 2) the lowest angle between trajectories.

### 3.4 Validation

#### 3.4.1 Manual versus automated approaches

We compare entry and target point lateral shift (LE and LT, respectively) and angle metrics computed automatically to those computed manually by a researcher at the National Hospital of Neurology and Neurosurgery (London, UK) with significant experience in clinical neuroscience. The full pipeline of about 10 electrodes takes over an hour to execute manually, on average. Two implanted trajectory approaches (manually computed in MATLAB) are validated: a) bolt axis (A1), and b) line of best fit of most superficial contacts outside of the bolt within a 20 mm threshold (A4). Note for either approach TP error is computed at the most distal contact, and hence is equivalent for A1 and A4.

Two points on the longitudinal bolt axis, i.e. bolt head and pivot point, and electrode contacts are manually marked via visual inspection of the icCT co-registered to the navCT on the neuronavigation system. EP location is identified by inspection on the scalp surface through the inserted bolt. Bolt axis and line-of-best-fit implanted trajectories are computed in MATLAB using bolt head and pivot point, and contact positions, respectively.

#### 3.4.2 Evaluation of trajectory estimation

There is no consensus in the literature of how best to estimate EP and angle errors with the majority of previous studies reporting only TP errors (Table 1). In this paper, we evaluate common choices to compute accuracy metrics including: implantation trajectory (bolt axis and line of best fit), EP definition (scalp or skull), and distance error measures. For entry and target point errors, we consider both lateral shift (LE and LT, respectively) and Euclidean distance (EE and ET, respectively).

#### 3.4.3 Statistical analysis

We use Box-Cox transformation (Box and Cox, 1964) of metric differences (manual minus automatic) and test for normality using D'Agostino-Pearson test. We then assess the degree of agreement between manual and automatic approaches using Bland-Altman analysis (Giavarina, 2015). The mean of the differences (μ) indicates the average bias of one method relative to the other whereas the limits of agreement (±1.96σ, i.e. within 95 % confidence interval) are used to quantify how well the methods agree for an individual implanted SEEG electrode. Statistical differences are assessed using non-parametric Wilcoxon signed-rank *W* and Mann-Whitney *U* tests for paired and group comparisons, respectively. Statistical analysis is done in SciPy (1.0.0). We use linear mixed models to investigate the effect of variables on accuracy metrics in R (3.5.2).

## 4 Results

**Fig. 3:**
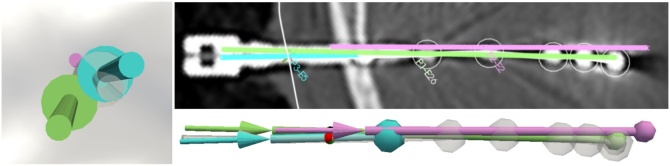
SEEG electrode trajectories. An example of electrode trajectories including: a) plan (pink), b) automatically segmented electrode (white translucent), c) automatically segmented bolt (blue), and d) line of best fit of contacts within 20 mm (green). Accuracy metrics related to this example are: LE is 2.107 mm, LT is 2.342 mm, and angle between line of best fit and plan is 1.987°.

Accuracy metrics of a total of 158 SEEG electrodes, of which 33 were implanted through temporal bone, were computed both manually and automatically. Table 1 shows metrics (average values across electrodes implanted per patient) computed automatically for lateral entry point (LE), lateral target point (LT) and angle difference errors between planned and implanted trajectories (line of best fit). Fig. 3 shows an example of a planned trajectory (pink), corresponding automated segmentation of the electrode (white) and trajectory computed from the bolt axis (blue) and line of best fit (green) to illustrate a case when metrics are higher than / similar to summative statistics (end of Table 1), yet it is difficult to assess implantation accuracy within this range of errors via visual inspection.

### 4.1 Manual versus automated

**Fig. 4:**
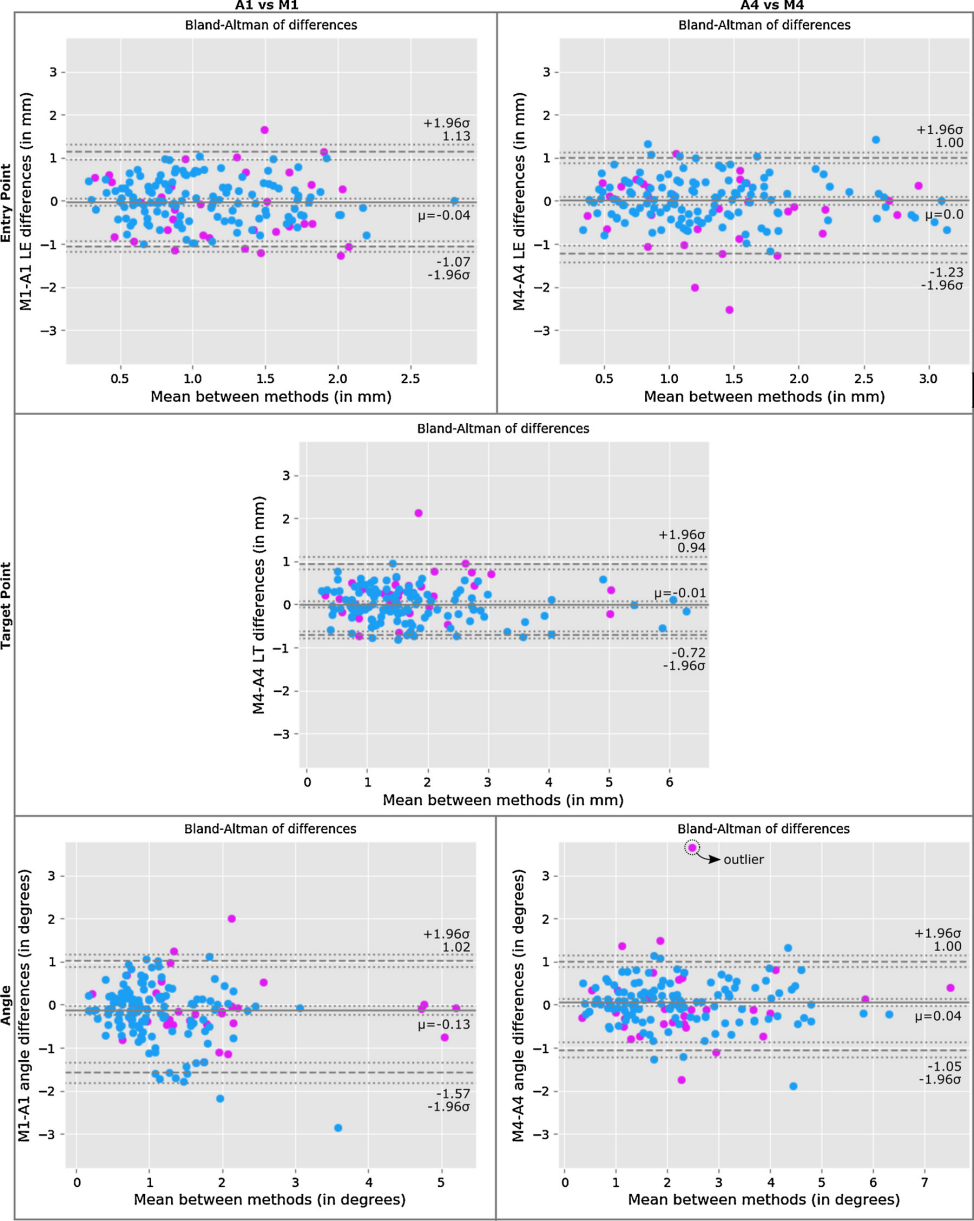
Validation of accuracy measures between manual and automated approaches (*left:* M1 vs A1; *right:* M4 vs A4). Bland-Altman plots of *top*) LE, *middle*) LT and *bottom*) angle difference between planned and implanted trajectories of 158 electrodes. Difference mean (solid line), -1.96 and +1.96 std. dev. (dashed lines), and their CI (dotted lines) are plotted following (Giavarina, 2015). Note for either approach TP error is computed at the most distal contact, and hence is equivalent for A1 and A4. Electrodes implanted through temporal bone are highlighted in pink.

Bland-Altman analysis shows a bias for smaller angles computed from the bolt axis (A1) with the automated approach having on average 0.13 higher angles. Similarly we found no statistically significant differences in the metrics between automated and manual approaches with the exception of angle differences when characterising the trajectory by bolt axis (*W = 4594.50; p = 0.0034*) (Table 2, **Supplemental Material**). These differences can be observed in electrodes implanted frontally targeting the insula and electrodes implanted through the parietal lobe targeting the cingulum (Table 3). We investigate the effect of registration errors between manual (neuronavigation system) and automated (NiftyReg) measurements finding no statistically significant changes in errors between manual and automated methods (see *Registration error* section in **Supplemental Material**). Due to previous reports (Cardinale et al., 2013), we investigate whether there is an effect of electrodes implanted through temporal bone (highlighted in pink colour in Fig. 4) on the metrics. Fixed effects included electrode length, maximum contact displacement and the interaction of approaches (i.e. manual or automatic) with implanted trajectory (i.e. bolt axis or LFB) whereas random effect included patients. We found that electrodes implanted in temporal bone had an effect on angle metrics (*χ^2^* = 20.57(4), *p < 0.001*), increasing it by 0.56° ±0.19 (std. error), with no effect on other metrics. Overall, electrode length has an statistically significant effect on the EP, TP and angle metrics of -0.01 mm, 0.02 mm and -0.011°, respectively, regardless of the approach used to compute the metrics. However, we found no statistically significant effect of length on metrics between manual and automated approaches.

**Table 2:** Accuracy metrics per patient. Mean values across electrodes (first row) and across electrodes stratified by temporal and non-temporal bone (second row).

Case	Number of electrodes	Lateralisation	Length (mm)	Skull Thickness (mm)	Max. Contact Displacement (mm)	Metrics		
						LE (mm)	LT (mm)	Angle (degrees)
1	8	right	38.4	6.3	0.69	1.3	1.38	2.13
	02-Jun		33.1 / 40.1	3.44 / 7.26	1.11 / 0.55	1.35 / 1.28	1.00 / 1.50	1.89 / 2.21
2	11	left	33.03	8.8	0.8	1.71	1.32	2.51
	01-Oct		41.3 / 32.1	3.16 / 9.42	1.02 / 0.78	0.84 / 1.81	1.39 / 1.31	3.50 / 2.40
3	13	right	30.8	7.26	0.78	1.42	1.43	2.21
	03-Oct		40.0 / 28.0	4.20 / 8.18	0.66 / 0.82	0.66 / 1.65	0.58 / 1.69	1.14 / 2.53
4	13	left	36.2	7.87	0.58	1.19	1.24	1.66
	01-Dec		45.7 / 35.44	4.46 / 8.16	0.50 / 0.58	0.60 / 1.24	1.17 / 1.24	1.95 / 1.64
5	9	right	33.1	7.38	0.93	1.37	1.58	2.2
	01-Aug		44.2 / 31.7	2.63 / 7.98	2.0 / 0.80	0.68 / 1.45	1.84 / 1.55	2.45 / 2.17
6	10	right	31.8	9.14	0.91	1.63	2.82	3.58
	04-Jun		30.1 / 32.9	4.76 / 12.05	0.83 / 0.96	1.94 / 1.42	1.67 / 3.59	2.33 / 4.41
7	10	left	42.8	5.91	0.77	1.65	2.26	2.21
	03-Jul		34.7 / 46.2	2.39 / 7.41	0.75 / 0.78	2.35 / 1.35	1.63 / 2.53	1.52 / 2.50
8	12	right	32.7	7.01	0.85	1.54	1.82	2.19
	02-Oct		36.5 / 31.9	3.23 / 7.76	0.63 / 0.89	2.15 / 1.42	1.41 / 1.90	2.28 / 2.17
9	7	left	41	5	0.91	0.96	1.48	2.4
	03-Apr		38.3 / 43.1	2.96 / 6.53	0.94 / 0.88	0.94 / 0.97	1.51 / 1.46	2.49 / 2.33
10	9	right	44.2	9.07	0.67	1.03	1.52	1.52
	02-Jul		40.1 / 45.4	4.37 / 10.42	0.54 / 0.71	1.80 / 0.81	1.54 / 1.51	0.99 / 1.66
11	12	right	40.1	8.09	0.67	1.33	1.61	1.32
	02-Oct		44.7 / 39.2	4.79 / 8.75	0.36 / 0.73	1.29 / 1.34	0.86 / 1.76	1.23 / 1.33
12	12	left	38.8	6.58	0.69	1.14	1.2	1.9
	03-Sep		36.7 / 39.5	2.55 / 7.92	0.79 / 0.65	1.19 / 1.13	2.25 / 0.85	3.07 / 1.5
13	13	left	38.5	7.1	0.71	0.95	1.82	2.08
	01-Dec		44.2 / 38.0	3.45 / 7.40	0.77 / 0.71	0.46 / 0.99	2.70 / 1.74	2.82 / 2.02
14	11	left	41.1	10.26	0.84	1.35	2.34	2.74
	03-Aug		37.4 / 42.6	5.14 / 12.17	0.82 / 0.85	2.02 / 1.11	2.69 / 2.20	3.29 / 2.54
15	9	left	38.6	5.23	0.91	1.09	2.17	2.87
	02-Jul		31.9 / 40.5	3.95 / 5.60	1.28 / 0.81	1.66 / 0.93	2.45 / 2.09	4.14 / 2.51
**Total**	**158**		**37.2**	**7.48**	**0.77**	**1.31**	**1.72**	**2.21**
	**33 / 125**		**37.3 / 37.18**	**3.76 / 8.46**	**0.83 / 0.76**	**1.46 / 1.28**	**1.64 / 1.75**	**2.29 / 2.18**

**Table 3:** Statistical significant differences of accuracy metrics per anatomical region between manual (M) and automated (A) approaches. Each cell, indexed by entry point and target point lobes/regions, indicates the number of electrodes (*n*) and metrics (*EP*, *TP*, *Angle*) with statistical differences at *p< = 0.05* (*) and *p< = 0.01* (**) significant levels of a Wilcoxon signed-rank test (*W*). Mean (standard deviation) of bolt axis (M1/A1) and line of best fit (M4/A4) approaches is shown for each metric within square brackets. A cell containing a hyphen (-) indicates that there were no electrodes found (EP-TP) in our study.

	Target Point (lobes and regions)							
		*frontal*	*central*	*temporal*	*parietal*	*occipital*	*insula*	*cingulum*
*Entry Point (lobes)*	*frontal*	n = 31	–	n = 5	–	–	n = 8 Angle: W = 1.0 (p = .02) * [M1 = .62 (.19); A1 = 1.16 (.63)]	n = 28
	*central*	–	n = 2	n = 5	n = 2	–	n = 4	n = 5 Angle: W = 0.0 (p = .04) * [M4 = 1.54 (.5); A4 = 1.01 (.38)]
	*temporal*	–	–	n = 40 TP: W = 218.5 (p = .01) ** [M4 = 2.0 (1.13); A4 = 1.8 (1.11)]	–	n = 4	n = 2	n = 2
	*parietal*	–	–	–	n = 4	–	n = 1	n = 14 EP: W = 17.0 (p = .03) * [M1 = 1.16 (.63); A1 = .95 (.66)] TP: W = 16 (p = .02) * [M4 = 2.42 (1.9); A4 = 2.66 (1.75)] Angle: W = 10.0 (p = .01) ** [M1 = .88 (.56); A1 = 1.56 (1.09)]
	*occipital*	–	–	–	–	–	–	n = 1

### 4.2 Evaluation of trajectory estimation

#### 4.2.1 Bolt Axis versus line of best fit

Bland-Altman analysis shows that, on average, there is a bias of 0.21 mm for LE and 0.86° for angle differences with higher errors reported when using the line of best fit (A4) compared to the bolt axis (A1) (Fig. 2 bottom, **Supplementary Material**). We found no effect of electrodes implanted through the temporal bone on metrics when considering maximum contact displacement. We evaluated two more line-of-best-fit approaches to characterise an implanted electrode trajectory using the position of two (A2) or three (A3) most proximal contacts (see *Line-of-best-fit approaches* section in **Supplemental Material**). Overall, we observed that metrics for A1 (bolt axis) have the lowest mean values and standard deviations, followed by A4 (Table 1, **Supplemental Material**).

#### 4.2.2 Entry point surface

We investigated whether there is a bias in the entry point metric (LE) for different EP surfaces, considering scalp versus skull on T1, navCT and icCT (see *Entry Point Surface* section in **Supplemental Material**). When characterising an implanted trajectory based on bolt axis (A1), we found no statistical difference between skull-based and scalp-based LE metrics. However, for A4 the scalp-based measures were on average 0.16 mm (*σ = 0.21*) higher than skull-based measures (*W = 1397.5; p < 0.001; R = 0.96*). The differences between scalp-based and skull-based metrics are higher for electrodes implanted through the temporal bone (*μ = 0.27; σ = 0.27*) compared to other electrodes (*μ = 0.13; σ = 0.18*). The differences between temporal and non-temporal electrodes were also statistically significant (*U = 1424.0; p < 0.001*). Moreover, when defining EP at level of scalp, we found that LE had the highest error using the icCT (*μ = 0.25* mm) image compared to LE metrics computed using T1/navCT (*μ = 0.19* mm), a difference which was statistically significant (see Table 3, **Supplemental Material**).

#### 4.2.3 Lateral shift versus euclidean distance

**Fig. 5:**
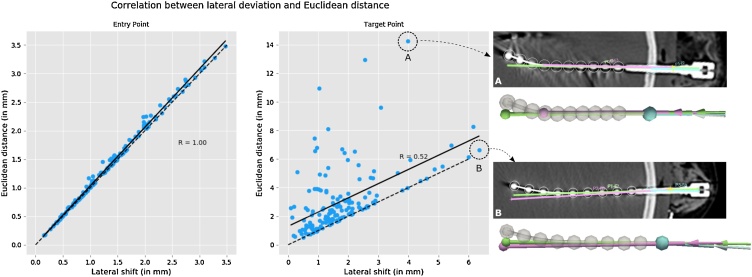
Correlation between metrics based on lateral shift (LE and LT) and Euclidean distance (EE and ET). Two examples related to TP metrics are highlighted, with one example showing how lateral shift is unable to capture errors in insertion depth of implanted trajectory (in green) compared to planned trajectory (in pink).

We investigated the correlation between lateral and Euclidean-based metrics (Fig. 5). Metrics related to EP are highly correlated *ρ = 1.0*, as both metrics are computed at the intersection of the planned and implanted trajectories with the scalp mesh. However, lateral shift metrics related to TP do not capture errors of electrode insertion depth (with respect to the plan), whereas Euclidean distance metrics capture these differences. This is reflected by higher variability between distance metrics and lower correlation when fitting a least squares regression model (*ρ = 0.52*). This variability, by comparison to EP distance metrics, is the result of differences in depth implantation with respect to the plan. Two example electrodes are highlighted: A) the metric based on Euclidean distance reports a much higher error compared to the lateral shift as the implanted trajectory was inserted further/shorter than initially planned), and B) both metrics report similar values as insertion depth was similar.

## 5 Discussion

Although computer-assisted surgical planning algorithms have been proposed for trajectory planning (De Momi et al., 2014; Sparks et al., 2017a, b), there have not been reports of automated approaches for quality assessment of SEEG electrode implantation. The computation of surgical metrics related to SEEG electrode implantation is key for quality assessment and to understand reasons for suboptimal outcomes. Here we propose and validate an automated method to compute metrics in relation to planning, including metrics based on lateral shift and Euclidean distance for a) EP, b) TP and c) angle difference. We also investigate the differences in metrics when using different methods to characterise electrode trajectories and entry points.

### 5.1 Validation of metrics between automated and manual approaches

The manual computation of accuracy metrics used to validate our automated approach consisted of few stages that required access to three different systems (Stealth, Matlab, and PACS) and high levels of concentration for processing data and moving it across systems. We therefore highlight the benefits of automated computation of metrics as a fast and reliable method to assess the quality of electrode implantation based entirely on medical images. We found no systematic differences between manual and automated approaches and the variability of their differences (shown by their limits of agreement) is similar across metrics and approaches (near 1 mm or 1° error), with the exception of a small bias towards higher angular deviations when using characterising the implanted trajectory with the automated bolt axis approach. This suggests that the line of best fit is more robust to inaccuracies of automatic segmentation of bolts and contacts positions by considering more points to construct the trajectory. Manual and automated approaches indicate that angle deviations are slightly higher on electrodes implanted through temporal bone than through other skull regions. This is consistent with previous findings (Cardinale et al., 2013) in which temporal regions are the most prone to drill deviation during surgery caused by the thinness and tridimensional curvature of the temporal bone. We verified registration differences (introduced by using two distinct algorithms, i.e. neuronavigation system and NiftyReg) have no effect on the observed differences in metrics.

### 5.2 Recommendations for reporting metrics

#### 5.2.1 Entry point surface

We observed a bias in scalp-based EP metrics compared to those that use a skull surface as a reference. Therefore, studies reporting EP deviations should detail how the EP is computed, although entry points defined on the skull surface are suggested. We found that entry points defined on the scalp surface, generated from a post-implantation CT image (icCT), introduce bias in trajectory angles caused by changes in scalp thickness as a response to scalp opening during implantation. When using a scalp to define the EP, a pre-operative image should be used to avoid biases in changes in scalp thickness.

#### 5.2.2 Lateral shift versus Euclidean distance

Correlation between distance metrics based on lateral and Euclidean distance unsurprisingly indicate lateral shift is unable to capture insertion depth errors. Studies reporting lateral errors should include a measure of insertion depth error to allow for comparison to studies which report Euclidean distance. When comparing SEEG implantation accuracy with different positioning systems (i.e Vertek™, frame, and robotic systems), errors in insertion depth may be irrelevant for measuring the accuracy of the positioning device. In these cases lateral shift may be the preferred metric as the depth component is controlled by the surgeon, whilst other factors are dependent on the implantation technique employed.

#### 5.2.3 Bolt Axis versus line of best fit

Our results suggest that the differences of metrics amongst the four approaches may be partially explained by displacement of the electrode from the desired rigid trajectory. These displacements may be caused by a combination of the following reasons: a) electrode bending (we observe a medium-to-large correlation between bolt axis and a line of best fit), b) differences in the position of contacts between manual and automated SEEG electrode segmentation (with reported mean average error of 0.38 mm, *σ = 0.24* (Granados et al., 2018)), and c) image co-registration errors (icCT-to-navCT Sorensen-Dice coefficient of skull: μ *= 92.23* and μ *= 84.45* using NiftyReg and neuronavigation system, respectively). A line of best fit (A4) is more robust to contact displacement compared to other line-of-best-fit approaches that uses only two or three contacts to estimate an implanted trajectory. These results indicate that the choice of line of best fit has an effect on EP and angle deviation metrics. While bolt axis might be a preferred trajectory representation (showing higher precision than all line of best fit approaches), although uncommon, any movement of the bolt after implantation (and before the icCT is taken), which is more likely to occur in temporal lobe trajectories in view of the thin skull bone overlaying the temporal lobe, giving less secure bolt anchorage, would result in inaccurate metrics. Studies reporting an implanted electrode trajectory should state clearly how trajectories are computed (i.e. bolt axis, line of best fit). If a line of best fit is preferred, a measure of contact displacement from a rigid trajectory (bending) of the points considered should be indicated.

### 5.3 Translation to other centres

Throughout our manuscript, we made our best to fully and clearly describe our methodology to facilitate its implementation by other centres. After pre- and post-implantation images are aligned into the same space (co-registered) and electrodes are (automatically) identified from CT images, i.e. position of bolts and contacts, the computation of accuracy metrics between planned and implanted trajectories is straightforward. However, we acknowledge that retrieving the planned trajectories is software-specific and is something each centre/manufacturer would need to determine to use this method. More importantly, we encourage other centres to keep in mind the recommendations proposed above when reporting accuracy metrics to facilitate comparison across systems that eventually can result in guidelines that are generalisable to different implantation techniques and types of electrodes.

## 6 Conclusions and future work

We presented an automated approach of SEEG electrode implantation accuracy assessment within a platform that supports all stages of electrode implantation. We provide recommendations for computing metrics in future studies: (a) report Euclidean distance or lateral shift with depth error information, (b) include both EP and TP localisation errors, (c) state clearly how EP position is identified, and (d) provide details of how trajectories are estimated. We highlight that our automated approach runs in a matter of seconds after automated electrode segmentation, is useful to avoid errors that may appear by doing tasks manually, guarantee consistency of a protocol, and avoids human bias. With the advent of robotic systems for SEEG electrode implantation, this research is relevant to automatically quantify accuracy of implantations (Vakharia et al., 2018). Our work is limited in a number of ways. Although we investigated the types of metrics reported in previous studies, this work is not a systematic review and the number of studies covered is not exhaustive. The data included in this study are from one centre using only Ad-Tech SEEG electrodes and might not be representative of other techniques and systems used. We envisage future work reporting variance due to factors affecting the accuracy of an implanted electrode, and the use of different navigation systems and types of electrodes with larger number of patients to provide guidelines of computing and reporting electrode implantation accuracy metrics.

## Ethical approval

All data were evaluated retrospectively. All studies involving human participants were in accordance with the ethical standards of the institutional and/or national research committee and with the 1964 Helsinki declaration and its later amendments or comparable ethical standards.

## Informed consent

For this type of study formal consent is not required.

## CRediT authorship contribution statement

**Alejandro Granados:** Conceptualization, Methodology, Software, Writing - original draft, Validation, Formal analysis, Writing - review & editing, Visualization. **Roman Rodionov:** Conceptualization, Methodology, Software, Investigation, Writing - review & editing. **Vejay Vakharia:** Conceptualization, Methodology, Investigation. **Andrew W. McEvoy:** Investigation. **Anna Miserocchi:** Investigation. **Aidan G. O'Keeffe:** Formal analysis. **John S. Duncan:** Supervision, Resources, Writing - review & editing, Project administration, Funding acquisition. **Rachel Sparks:** Methodology, Supervision, Writing - review & editing, Visualization. **Sébastien Ourselin:** Supervision, Resources, Project administration, Funding acquisition.

## Declaration of Competing Interest

The authors declare that they have no conflict of interest.

## Figures and Tables

**Fig. A1:**
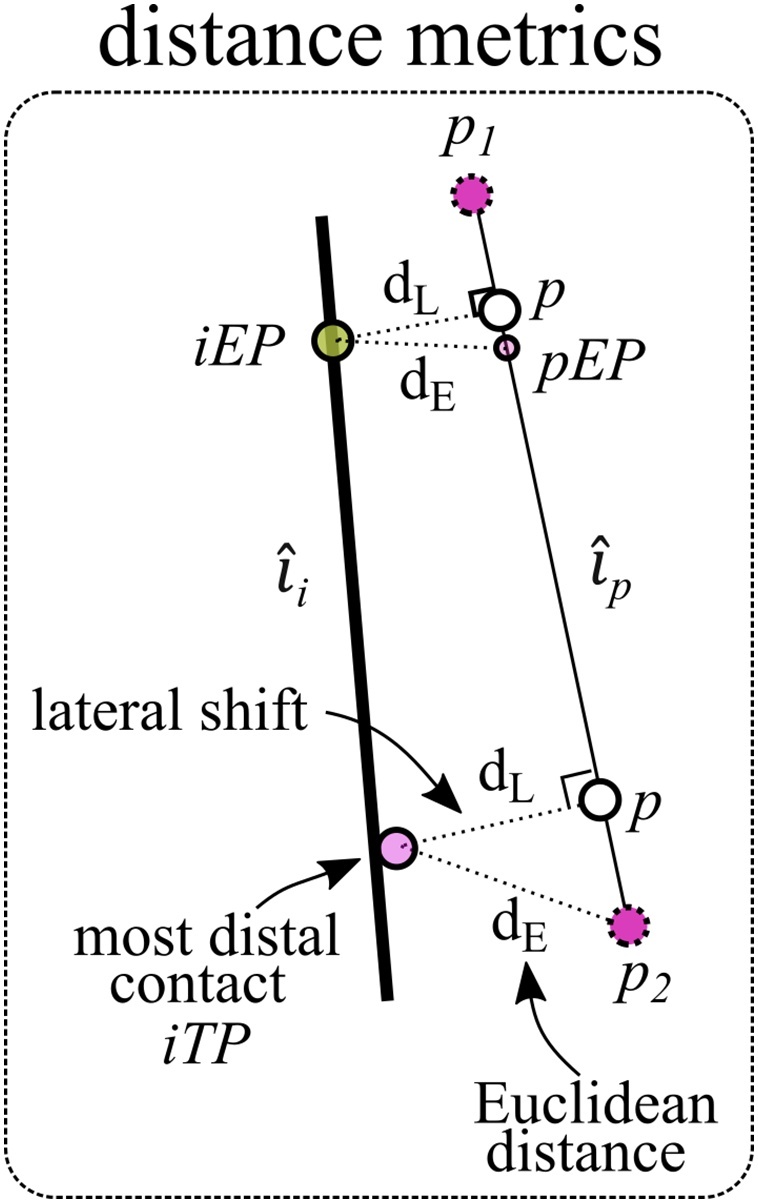
Lateral shift and Euclidean distance.

## Appendix A Accuracy Metrics

*Euclidean norm* (Eq. A.1) is used to define the shortest distance *d* between points *p* and *q* in 3D space.

(A.1) $$d\left(p,q\right)=\left\|p-q\right\|=\left\|q-p\right\|=\sqrt{{\left(p_{x}-q_{x}\right)}^{2}+{\left(p_{y}-q_{y}\right)}^{2}+{\left(p_{z}-q_{z}\right)}^{2}}$$

*Lateral Shift*. We compute lateral shift, $d_{L}\left(.,.\right)$ between a point $i\in \hat{l_{i}}$ and the line $\hat{l_{p}}$, defined between the points *p_1_* and *p_2_*. We computed $d_{L}\left(i,p\right)=\left\|i-p\right\|$ where *p* is defined as the point on $\hat{l_{p}}$ that satisfy the criteria $\left(i-p\right)∙\left(p_{2}-p_{1}\right)=0$ (See Fig. A1). Following the above, we compute lateral shift of entry point (LE) and use the most distal contact (*i = iTP*) to compute the lateral shift of target point (LT).

*Euclidean Distance*. Euclidean distance is the distance $d_{E}\left(.,.\right)$ of a straight line between two points and is computed as $d_{E}\left(i,p_{2}\right)=\left\|i-p_{2}\right\|$ (See Fig. A1 right panel). For each electrode, we then compute Euclidean distances of entry point (EE) and, similar to lateral shift, we use *i = iTP* to compute the Euclidean distance of target point (ET).

*Angle difference*. The angle difference θ (in degrees) between a planned ($\hat{l_{p}}$) and implanted ($\hat{l_{i}}$) trajectory is computed as $\theta =\text{arctan}\frac{\left\|\hat{l_{p}}\times \hat{l_{i}}\right\|}{\hat{l_{p}}∙\hat{l_{i}}}*\frac{180}{\pi }$.

## Appendix B Line of best fit

We find the line of best fit for *M*, a matrix where each row *n* (*M_n,:_*) corresponds to one of *N* points along the electrode, comprising electrode contacts and/or the bolt (Fig. 2). We first compute the centroid point $\bar{c}$ of *M* (Eq. B.1a). We then compute *D*, a matrix describing the contact variation, by subtracting $\bar{c}$ from *M* (Eq. B.1b). We compute a singular value decomposition (SVD) of *D* (Eq. B.1c), where $U\in R^{N\times N}$ and $V\in R^{3\times 3}$ are the left and right orthogonal singular vectors of *D*, respectively. The diagonal entries of $\text{Σ}\in R^{N\times 3}$ are the singular values ($\sigma_{i}$) of *D*, arranged in descending order. The line of best fit of an SEEG electrode implanted trajectory is then defined as $\hat{l_{i}}=\bar{c}+v_{1}$, where $v_{1}$ is the first column of *V* corresponding to the highest singular value in Σ.

(B.1a) $$\bar{c}=\frac{1}{N}\sum_{n=1}^{N}M_{n,:}$$

(B.1b) $$D=M-\bar{c}$$

(B.1c) $$D=U\text{Σ}V^{T}$$
