# Supplementary material for: Automated computation and analysis of accuracy metrics in stereoencephalography
Source: J Neurosci Methods. 2020 Jul 1;340:108710. doi: 10.1016/j.jneumeth.2020.108710 (PMC7456795; doi:10.1016/j.jneumeth.2020.108710)
Supplement: Supplementary file 1 [file mmc1.pdf]

Automated computation and analysis of accuracy metrics in stereoencephalography
Supplemental Material

Alejandro Granados[a,*], Roman Rodionov[b], Vejay Vakharia[b], Andrew W. McEvoy[b], Anna Miserocchi[b], Aidan G. O'Keeffe[d], John S. Duncan[b,c], Rachel Sparks[a], Sébastien Ourselin[a]

[a]*School of Biomedical Engineering and Imaging Sciences, King's College London, UK*
[b]*National Hospital of Neurology and Neurosurgery, London, UK*
[c]*Dept of Clin and Experim Epilepsy, UCL Queen Square, Inst of Neurol, UK*
[d]*Dept of Statistical Science, University College London, UK*

Abstract

Implantation accuracy during neurosurgical interventions, such as placement of intracranial SEEG or deep brain stimulation electrodes, is necessary to ensure safety and efficacy. Typically, metrics are computed by visual inspection which results in a process that is tedious, prone to inter-/intra-observer variation, and difficult to replicate across sites. Neither the choice of metrics nor the possible errors that could affect such metrics have been investigated. We present an automated approach for computing the accuracy of implantation of intracerebral electrodes, and the potential errors. This supplemental material provides further information about the potential sources of errors including line-of-best-fit approaches, registration errors, electrode bending and definition of entry point (EP) surface.

Keywords: Epilepsy, SEEG, Accuracy, Metrics

1. Line-of-best-fit approaches

We evaluated four methods to compute line of best fit to accurately estimate the implanted electrode trajectory (Fig. 1): line of best fit computed using bolt head position and pivot point at skull level (A1: bolt axis), two most proximal contacts (A2), three most proximal contacts (A3), and most proximal contacts within a distance set to 20 mm (A4). Metrics related to TP, i.e. LT and ET, are not affected by the line of best fit as deviation is measured using only the position of the most distal contact (iTP). Overall, we observed that metrics for A1 (bolt axis) have the lowest mean values and standard deviations, followed by A4 (Table 1).

*Corresponding author
Email address: alejandro.granados@kcl.ac.uk (Alejandro Granados)

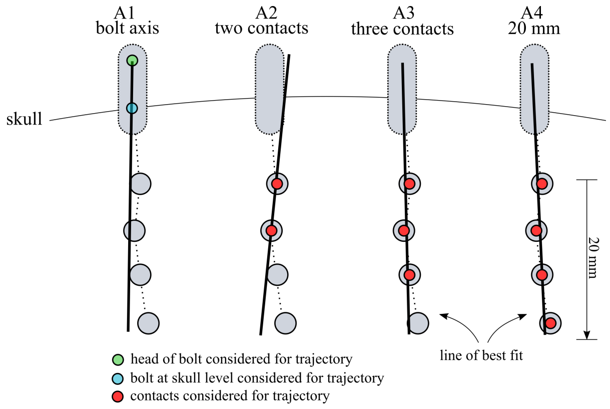

Figure 1: Four line-of-best-fit approaches: A1) bolt axis (i.e. bolt head to pivot point at level of skull), A2) two most proximal contacts, A3) three most proximal contacts, and A4) N contacts that are within a threshold distance $d_\lambda = 20$ mm.

Table 1: Comparison of accuracy metrics based on trajectory estimation methods.

**Metrics**	**Line-of-best-fit approaches**			
	**A1**	**A2**	**A3**	**A4**
**EP lateral shift**	$\mu = 1.1$ $\sigma = 0.58$	$\mu = 1.55$ $\sigma = 0.82$	$\mu = 1.38$ $\sigma = 0.77$	$\mu = 1.32$ $\sigma = 0.72$
**Angle Difference**	$\mu = 1.33$ $\sigma = 0.95$	$\mu = 2.75$ $\sigma = 1.61$	$\mu = 2.34$ $\sigma = 1.29$	$\mu = 2.21$ $\sigma = 1.33$

2. Registration Error

We assessed the effect differences of image co-registration between pre- (navCT) and post-operative (icCT) CT images using StealthMerge™ on the neuronavigation system and using NiftyReg [1] within the EpiNav™ workflow, had on the computed metrics as follows. We computed a rigid transformation matrix (rotation and translation) to minimise the Euclidian distance between electrode contacts points identified on the neuronavigation system and automatically within EpiNav™. We applied this transformation to the contacts marked manually, to better align these points in the navCT space and then recompute trajectory metrics. The registration correction decreased the mean average error of the contact position from $\mu = 0.69$ ($\sigma = 0.21$) mm to $\mu = 0.28$ ($\sigma = 0.17$) mm (Fig. 2). Table. 2 shows that there were no statistically significant differences after accounting for registration errors between manual (neuronavigation system) and automated (NiftyReg) measurements.

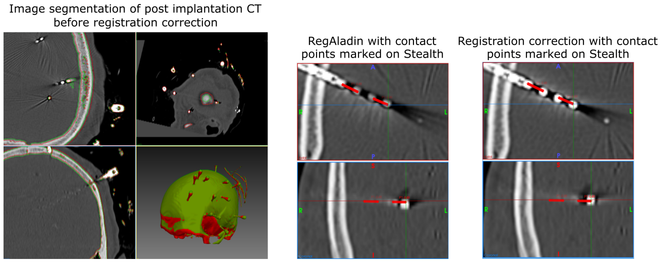

Figure 2: Registration correction example. *Left:* Image segmentation of resulting co-registered post-implantation CT from image co-registration on the neuronavigation system (in red) and RegAladin (in green) before registration. *Right:* Manually marked contact points overlaid on co-registered post-implantation CT from RegAladin before and after registration correction.

Table 2: Comparison between manual (M) and automated (A) computation of accuracy metrics of two implanted trajectory approaches: a) bolt axis (M1 vs A1) and b) line of best fit (LBF) of contacts within a 20 mm threshold (M4 vs A4). Wilcoxon signed-rank test (W) and statistics of differences ($M - A$) are reported. There were no statistically significant differences after accounting for registration errors between manual (neuronavigation system) and automated (NiftyReg) measurements (see Sec. 3.1).

	**Without additional registration**		**With additional registration**
**Implanted Trajectory**	**Bolt axis (A1 vs M1)**	**LBF of 20.0 mm (A4 vs M4)**	**LBF of 20.0 mm (A4 vs M4)**
**Entry point lateral shift**	$W = 6008$ ($p = 0.64$)<br>$\mu = -0.04$ ($\sigma = 0.56$)<br>$CI = [-1.07, 1.13]$	$W = 6061.50$ ($p = 0.70$)<br>$\mu = 0.0$ ($\sigma = 0.58$)<br>$CI = [-1.23, 1.00]$	$W = 5885.00$ ($p = 0.49$)<br>$\mu = -0.03$ ($\sigma = 0.54$)<br>$CI = [-1.10, 1.03]$
**Target point lateral shift**	$W = 5944.00$ ($p = 0.56$)<br>$\mu = -0.01$ ($\sigma = 0.43$)<br>$CI = [-0.72, 0.94]$		$W = 5394.00$ ($p = 0.12$)<br>$\mu = 0.05$ ($\sigma = 0.39$)<br>$CI = [-0.71, 0.81]$
**Angle difference**	$W = 4594.50$ ($p = 0.0034$)<br>$\mu = -0.13$ ($\sigma = 0.67$)<br>$CI = [-1.57, 1.02]$	$W = 5715.00$ ($p = 0.39$)<br>$\mu = 0.04$ ($\sigma = 0.60$)<br>$CI = [-1.05, 1.00]$	$W = 5831.00$ ($p = 0.52$)<br>$\mu = 0.04$ ($\sigma = 0.60$)<br>$CI = [-1.14, 1.22]$

3. Bolt Axis versus Line of best fit

We characterised electrode bending as measured by the maximum difference between predicted contact location from the original plan and measured contact location on the post operative scan. A linear mixed effect model comprising maximum contact displacement and trajectory approach (A1 and A4) as fixed effects and patient as the random effect was fit to the data. This model indicated that maximum contact displacement have an effect ($p < 0.001$) on LE ($0.4mm$ increases) and angle ($1.45°$) metrics. This model also found the choice of approach (bolt (A1) versus line of best fit (A4)) affected the metrics, with line of best fit (A4) increasing LE (0.05 mm; $p < 0.01$) and angle ($0.22°$; $p < 0.001$). We observe more variability in angle metrics using a line of best fit (A4) compared to a bolt axis (A1) (Fig. 3 top). There is an increase of variability ([-3.62, 1.79]) of angle differences between A1 and A4 as the size of the data increases and a trend where A1 is bigger than A4 for low mean values between methods, but A1 is lower than A4 for high mean values (mean values are shown in the horizontal axis in the Bland-Altman plots in Fig. 3 bottom).

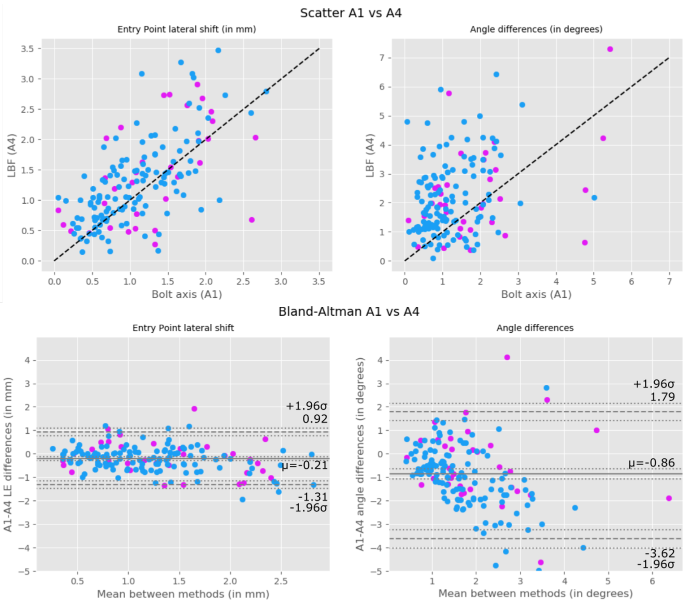

Figure 3: Comparison between bolt axis (A1) and line of best fit (A4). Scatter *top* and Bland-Altman *bottom* plots of LE and angle differences between A1 and A4. Electrodes implanted through temporal bone are highlighted in pink.

4. Entry Point Surface

EP errors are calculated from the point planned and implanted trajectories ($\hat{l}_i$ or $\hat{l}_p$) intersects a surface mesh $\mathbb{S}$. Angle error θ and distance l from the pivot point (at the skull level) will introduce bias in this metric, namely $\epsilon_{surface} = l * cos(\theta)$. To estimate this bias, we computed LE for different surface meshes: skull from navCT (reference), scalp from T1, scalp from navCT, and scalp from icCT. We found that the differences of LE of a scalp from T1 or navCT, with respect to the LE using the skull, are similar and have the lowest error ($\mu = 0.19$; $\sigma = 0.17$) (Table 3). The scalp from icCT has the highest error and is statistically significant different to the errors observed for the scalp from T1 ($p = 0.0105$) and the scalp from navCT ($p = 0.0033$), where p-values have been adjusted following Bonferroni correction (Fig. 4). It should be noted that outliers were observed in electrodes implanted in the temporal bone, where the skull is at its thinnest.

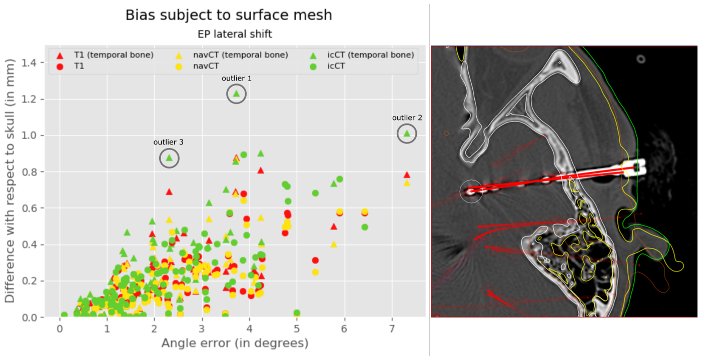

Figure 4: LE bias subject to surface mesh. *Left:* Scatter plot of angle error and distance from skull with entry points identified from T1 (red), navCT (yellow) and icCT (green). Electrodes implanted through temporal bone are plotted with triangles. Three outliers are highlighted. *Right:* Screenshot of electrode trajectory (planned and implanted) related to Outlier 1 outlining skull from navCT (white), and scalp from T1 (red), navCT (yellow) and icCT (green).

Table 3: EP surface errors with respect to skull (navCT) of different scalp surface meshes generated from: T1, navCT and icCT.

	**Image**	**Mean**	**Std. Dev.**	**Significance**
**Scalp**	**T1**	$\mu = 0.19$	$\sigma = 0.17$	T1 vs navCT: $p = 0.36$
	**navCT**	$\mu = 0.19$	$\sigma = 0.17$	T1 vs icCt: $p = 0.003$
	**icCT**	$\mu = 0.25$	$\sigma = 0.22$	navCT vs icCT: $p = 0.001$

References

[1] M. Modat, D. M. Cash, P. Daga, G. P. Winston, J. S. Duncan, S. Ourselin, Global image registration using a symmetric block-matching approach, J of Medical Imaging 1 (2).
